# The Anti-Inflammatory Effect of *Prunus yedoensis* Bark Extract on Adipose Tissue in Diet-Induced Obese Mice

**DOI:** 10.1155/2015/937904

**Published:** 2015-08-30

**Authors:** Hee Kang, Tae-Kyung Kwak, Bo-Geun Kim, Kyung-Jin Lee

**Affiliations:** ^1^Graduate School of East-West Medicine, Kyung Hee University, Yongin 446-701, Republic of Korea; ^2^Department of Herbology, College of Korean Medicine, Kyung Hee University, Seoul 130-701, Republic of Korea

## Abstract

Chronic, low-grade inflammatory responses occur in obese adipose tissue and play a crucial role in the development of insulin resistance. Macrophages exposed to high glucose upregulate the expression of SRA, a macrophage-specific scavenger receptor. The present study investigated whether *Prunus yedoensis* (PY) bark extract affects the inflammatory response and scavenger receptor gene expression observed in a diet-induced obesity model *in vivo*. Oral administration of PY extract significantly reduced fasting blood glucose levels without a change in body weight in mice fed a high fat diet for 17 weeks. PY extract significantly suppressed expression of inflammatory and macrophage genes such as tumor necrosis factor-*α*, interleukin-6, and F4/80 in epididymal adipose tissue. Among scavenger receptor genes, SRA expression was significantly reduced. The inhibitory responses of PY extract and its fractions were determined through evaluation of scavenger receptor expression in THP-1 cells. PY extract and its ethyl acetate fraction decreased the levels of SRA mRNA and phospho-ERK1/2 during monocyte differentiation. Our data indicate that the anti-inflammatory effects of PY extract and its downregulation of SRA seem to account for its hypoglycemic effects.

## 1. Introduction

Chronic, low-grade inflammatory responses are observed in obese and insulin-resistant individuals. In rodent obesity models, the upregulation of inflammation and macrophage-specific genes occurs in obese adipose tissue before insulin resistance develops [1]. It remains unclear how inflammatory signals are initiated in obese adipose tissue, but there is evidence that free fatty acids released by adipocytes stimulate the expression of chemokines such as monocyte chemoattractant protein-1 (MCP-1), which is originally expressed in macrophages and endothelial cells [2]. MCP-1 secreted by adipocytes recruits circulating monocytes to adipose tissue, thereby increasing the number of adipose tissue macrophages [3, 4]. Furthermore, when adipocytes are exposed to the proinflammatory cytokine tumor necrosis factor- (TNF-) *α*, the tyrosine kinase activity of the insulin receptor decreases, resulting in reduced uptake of glucose [5, 6].

Scavenger receptors such as SRA, CD36, and lectin-like oxidized LDL receptor-1 (LOX-1) mediate the recognition and internalization of polyanionic macromolecules [7]. SRA, which is mainly expressed in macrophages, contributes to important biological functions of macrophages, which clear unwanted cellular debris and metabolic products [7]. Excessive uptake of oxidized lipids through SRA or CD36 renders macrophages into lipid-laden foam cells, which subsequently die and release their lipids, the culprit responsible for necrotic, calcified plaques [8]. Macrophages exposed to high glucose upregulate the expression and activity of SRA [9]. Recently, the expression of SRA was reported to be positively associated with insulin resistance in obese, nondiabetic humans [10].

*Prunus yedoensis* (PY) bark is a component of Shim-Mi-Pa-Dok-San, a complex herbal formula used to treat chronic skin inflammatory disorders in Korea and China. It is also used as an antitussive and antiphlegm medicine in Japan. Previously, we identified prunetin in PY extract and demonstrated that oral administration of PY extract decreased serum levels of TNF-*α* and interleukin- (IL-) 6 upon intraperitoneal lipopolysaccharide (LPS) injection in mice [11]. Intraperitoneal administration of prunetin decreased the body weight and blood glucose levels in mice fed a high fat diet (HFD) [12]. This led us to hypothesize that PY extract may affect the chronic inflammatory responses observed in obese adipose tissue. In the present study, we examined the influence of PY extract on diet-induced obesity* in vivo*. Also, the effects of PY extract and its fractions were determined through evaluation of scavenger receptor expression in THP-1 cells.

## 2. Materials and Methods

### 2.1. Preparation of PY Extract and Its Fractions

*Prunus yedoensis* bark (Dongwoodang, South Korea) was identified and authenticated by Professor Choi Ho-Young, Department of Herbology, Kyung Hee University. A voucher specimen (number 2013-PY-80E) was deposited at the Laboratory of Herbal Immunology, Kyung Hee University. Briefly, 0.4 kg of ground PY was extracted in 80% aqueous ethanol with a heating mantle and reflux, twice for 2 h each, and filtered through filter paper. The extract was concentrated using a rotary evaporator and freeze-dried in vacuum. One gram of the extract was subjected to solvent fractionation using chloroform, ethyl acetate (EA), and water. Each fraction was also concentrated by evaporation and freeze-dried in vacuum. The yields of each fraction were 2.5%, 48%, and 43% for the chloroform, EA, and aqueous fractions, respectively.

### 2.2. Diet-Induced Obesity

Five-week-old male C57BL/6 mice were purchased from the Korean branch of Taconic, SamTaco (Osan, South Korea), and housed in a temperature- and humidity-controlled pathogen-free animal facility with a 12 h light-dark cycle at the Medical Center of Kyung Hee University Hospital. After acclimatization for 1 week, mice were divided into three groups (*N* = 10, each). Mice in group one were fed a regular chow diet (RCD) and mice in groups two and three were fed a HFD which derived 60% of its calories from fat (diet D12492, Research Diets Inc., USA). Mice were maintained on the diet for 17 weeks. Oral gavage was performed in group three (HFD/PY) with 100 mg/kg of PY extract daily throughout the entire experimental period. In our previous work, oral doses in the range of 50–250 mg/kg for seven consecutive days proved to be anti-inflammatory* in vivo* [11]. Due to the long administration period, the dose was determined based on the midrange of our previous results. Body weight was measured weekly. The animal protocol (KHMC-IACUC: 12-006) was approved by the Kyung Hee University Medical Center Institutional Animal Care and Use Committee, and mice were cared for according to the US National Research Council* Guide for the Care and Use of Laboratory Animals* (1996).

### 2.3. Gene Expression Analysis

Mice were anesthetized with ether and sacrificed at the end of the experiment. Epididymal white adipose tissue was collected and total RNA was isolated using the RNeasy Lipid Tissue Mini Kit (Qiagen, Germany) according to the manufacturer's instructions. Total RNA from THP-1 cells was extracted using the RNeasy Mini Kit (Qiagen). Synthesis of cDNA was performed with Superscript RT III (Invitrogen, USA), using 2 micrograms of total RNA and an oligo-(dT)_12–18_ primer (Invitrogen). Appropriately diluted cDNA was mixed with Power SYBR Green PCR Master Mix (Applied Biosystems, USA) and 3 pmol of primers. The primer sequences were as follows: mouse TNF-*α*: forward: 5′-CCCTCACACTCAGATCATCTTCT-3′ and reverse: 5′-GCTACGACGTGGGCTACAG-3′; mouse IL-6: forward: 5′-TAGTCCTTCCTACCCCAATTTCC-3′ and reverse: 5′-TTGGTCCTTAGCCACTCCTTC-3′; mouse F4/80: forward: 5′-TGACTCACCTTGTGGTCCTAA-3′ and reverse: 5′-CTTCCCAGAATCCAGTCTTTCC-3′; mouse MCP-1: forward: 5′-TTAAAAACCTGGATCGGAACCAA-3′ and reverse: 5′-GCATTAGCTTCAGATTTACGGGGT-3′; mouse GAPDH: forward: 5′-GGCATGGACTGTGGTCATGA-3′ and reverse: 5′-TTCACCACCATGGAGAAGGC-3′; human SRA: forward: 5′-GCAGTGGGATCACTTTCACAA-3′ and reverse: 5′-AGCTGTCATTGAGCGAGCATC-3′; human CD36: forward: 5′-GCCAAGGAAAATGTAACCCAGG-3′ and reverse: 5′-GCCTCT GTTCCAACTGATAGTGA-3′; human LOX-1: forward: 5′-CTCGGGCTCATTTAACTGGGA-3′ and reverse: 5′-AGGAAATTGCTTGCTGGATGAA-3′; and human actin: forward: 5′-GCAAATGCTTCTAGGCGGACTAT-3′ and reverse: 5′-TGTTTTCTGCGCAAGTTAGGTTT-3′. Amplification of cDNA was performed in triplicate using a StepOnePlus Real-time PCR system (Applied Biosystems). PCR conditions were set at an initial denaturation at 95°C for 10 min, followed by 40 cycles of 95°C for 15 s and 60°C for 1 min. Relative gene expression was determined using the standard curve method. Quantities of each gene were normalized to GAPDH or actin values.

### 2.4. Determination of Fasting Blood Glucose

Fasting blood glucose was determined at week 16. Blood samples were obtained from the tail vein after 16 hours of fasting, and glucose levels were measured using a portable glucose sensor (ACCU-CHEK Aviva, Roche Diagnostics, USA).

### 2.5. THP-1 Cell Experiments

THP-1 cells, a human monocytic cell line, were obtained from the Korean Cell Line Bank (Seoul, South Korea). Cells were maintained in RPMI medium (Hyclone, USA) with 10% fetal bovine serum (Hyclone), 25 mM HEPES, and 1% penicillin-streptomycin. To induce differentiation to macrophages, cells were treated with 100 nM PMA (Sigma, USA). For cell viability experiments, 4 × 10^4^ cells in 0.1 mL were plated per well in 96-well plates and cultured for 24 h and 48 h with PY extract and its fractions. PMA was added simultaneously. For gene expression analysis, 2 × 10^6^ cells in 2 mL were plated in 6-well plates for 48 h with PY extract (200 *μ*g/mL) or with the aqueous (100 *μ*g/mL), ethyl acetate (100 *μ*g/mL), or chloroform (10 *μ*g/mL) fractions thereof. For the extracellular signal-related kinase (ERK) 1/2 activation experiments, cells were pretreated with PY extract and its fractions for 1 h prior to exposure to PMA for 30 min.

### 2.6. Cell Viability Assay

Cell viability was determined using a Cell Counting Kit-8 assay (Enzo Life Sciences, USA).

### 2.7. Western Blotting

Cells were rinsed in cold PBS and then lysed on ice in RIPA buffer (50 mM Tris-HCl, pH 7.5; 150 mM NaCl; 1 mM EDTA; 20 mM NaF; 0.5% NP-40; and 1% Triton X-100) containing phosphatase inhibitor cocktail (Sigma) and Xpert protease inhibitor cocktail (GeneDEPOT, USA). After centrifugation at 13,000 ×g for 10 min, supernatants were collected, and protein concentrations were determined using the Bradford protein assay reagent (Bio-Rad, USA). The samples were separated on a 10% SDS-polyacrylamide gel and were transferred to polyvinylidene fluoride membranes. The membranes were blocked with 5% skim milk in Tris-buffered saline with 0.1% Tween 20 (TBST) for 1 h. The membranes were incubated with antibodies against phospho-ERK 1/2 (Cell Signaling Technology, USA) and GAPDH (Santa Cruz Biotechnology, USA) in 5% skim milk in TBST overnight at 4°C. The blots were washed with TBST and incubated for 1 h with anti-goat, anti-mouse, or anti-rabbit horseradish peroxidase-conjugated antibodies. Immunoreactive bands were visualized with EzWestLumi plus (ATTO, Japan) and read using a chemiluminescent analyzer system, EZ-Capture MG (ATTO).

### 2.8. HPLC

Samples were analyzed using a reversed-phase HPLC system (Shimadzu, Japan) with a Zorbax Eclipse XDB-C18 column (5 *μ*m × 4.6 mm × 250 mm) (Agilent Technologies, USA). Chromatography was performed at room temperature (RT) at a flow rate of 0.5 mL/min, and 10 *μ*L was analyzed for 110 min. The mobile phase consisted of 0.1% formic acid (A) and acetonitrile (B) in a ratio specified by the following binary gradient with linear interpolation: 0 min 20% B, 60 min 30% B, 70 min 60% B, and 100 min 70% B. The column eluent was monitored by UV spectrophotometry for absorbance at a wavelength of 280 nm. The contents of genistein, prunetin, and sakuranetin (Sigma) in chloroform fraction were quantified.

### 2.9. Determination of Total Phenolics and Flavonoids

Total phenolics were determined using gallic acid equivalent. 0.2 mL of each diluted fraction was mixed with 2.6 mL of deionized distilled water (DDW) and then incubated with 0.2 mL of Folin and Ciocalteu (FC) reagent (Sigma) for 5 min at RT. 2 mL of 7% Na_2_CO_3_ was added and then incubated for 90 min at RT. Absorbance was read at 750 nm and the concentration of total phenolics was calculated as gallic acid (Sigma) equivalent per gram of dry weight of fraction. Total flavonoids were determined as catechin equivalent. 0.5 mL of each diluted fraction was mixed with 3.2 mL of DDW. 0.15 mL of 5% NaNO_2_ was added and then, after 5 min, 0.15 mL of 10% AlCl_3_ was added. After 10 min, 1 mL of 1 M NaOH was added and read at 510 nm. The concentration of flavonoids was calculated as catechin (Simga) equivalent per gram of dry weight of fraction.

### 2.10. Statistical Analysis

Statistical evaluation was carried out using Student's* t*-test or one-way analysis of variance followed by the LSD test using IBM SPSS Statistics version 21.* P* values less than 0.05 were considered significant.

## 3. Results

### 3.1. PY Decreased Fasting Blood Glucose Levels but Not Body Weight in Mice Fed a HFD

**Figure 1:**
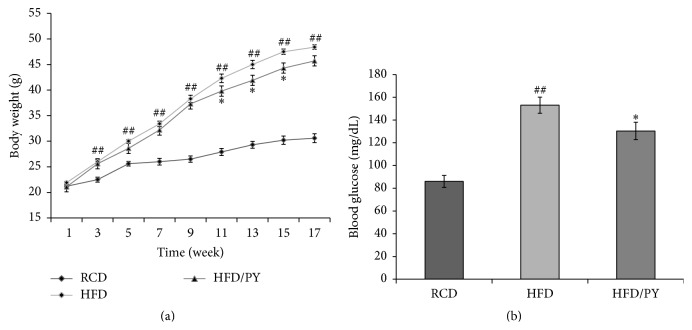
Effect of* Prunus yedoensis* (PY) bark extract on body weight and fasting blood glucose levels in mice fed a high fat diet. Mice were fed a regular chow diet (RCD) or high fat diet (HFD) for 17 weeks. PY extract (100 mg/kg) was orally administered to mice daily throughout the entire experimental period. (a) Changes in body weight were recorded over 17 weeks. (b) Fasting blood glucose levels were measured at week 16. *N* = 10 per group. ^##^ *P* < 0.005 versus RCD group, ^*∗*^ *P* < 0.05 versus HFD group.

The body weight of mice fed a HFD significantly increased to 48.4 ± 0.45 g, at week 17 (*P* = 0.000 versus RCD group), while that of mice on a RCD was 30.6 ± 0.85 g (Figure 1(a)). Relative to the HFD group, the body weight of the HFD/PY group was lower, at 45.7 ± 1.4 g, but this did not have statistical significance (*P* = 0.065 versus HFD group). We measured fasting blood glucose levels at week 16. The blood glucose level in the RCD group was 86.01 ± 5.23 mg/dL, and HFD feeding significantly increased this to 153.1 ± 7.05 mg/dL (*P* = 0.000 versus RCD group). PY extract significantly reduced fasting blood glucose by 15% to 130.4 ± 0.85 mg/dL (*P* = 0.024 versus HFD group) (Figure 1(b)).

### 3.2. PY Extract Suppressed the Upregulation of Inflammatory Markers in Epididymal Adipose Tissue in Diet-Induced Obesity

**Figure 2:**
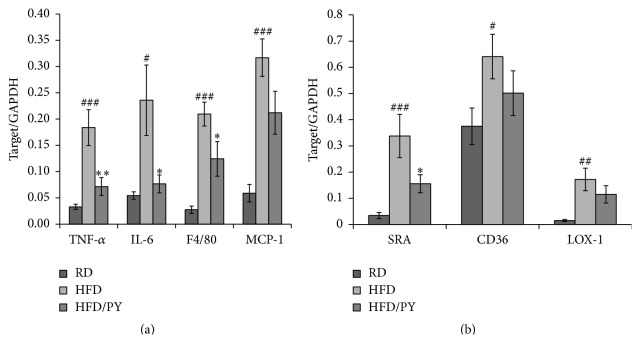
Effect of PY extract on inflammatory and macrophage gene expression in adipose tissue of mice fed a HFD. Adipose tissue was obtained at week 17. Quantitative PCR was used to measure the expression of TNF-*α*, IL-6, F4/80, and MCP-1 (a) and SRA, CD36, and LOX-1 (b). Gene expression was normalized to that of GAPDH. Data are expressed as mean ± SEM. *N* = 5 per group. ^#^ *P* < 0.05, ^##^ *P* < 0.01, and ^###^ *P* < 0.005 versus RCD group, ^*∗*^ *P* < 0.05 and ^*∗∗*^ *P* < 0.01 versus HFD group.

We evaluated whether any change in obesity-related inflammatory genes might have occurred. The genes tested here were TNF-*α* and IL-6, representative inflammatory cytokines; F4/80, a mature macrophage-specific surface protein; and MCP-1. TNF-*α* and IL-6 gene expression in the HFD group increased significantly, over 5.5- and 4.3-fold (*P* = 0.000 for TNF-*α*, *P* = 0.014 for IL-6 versus RCD group), respectively, relative to the RCD group (Figure 2(a)). PY extract significantly decreased the elevated levels of TNF-*α* and IL-6, by 60% (*P* = 0.004) and 70% (*P* = 0.017), respectively, compared to the HFD group. HFD feeding significantly increased the mRNA levels of MCP-1 and F4/80, by 5.4- and 7.6-fold (*P* = 0.001 for MCP-1, *P* = 0.000 for F4/80 versus RCD group), respectively (Figure 2(a)). Treatment with PY extract significantly suppressed the expression of F4/80 by 40% (*P* = 0.026) but did not suppress that of MCP-1 (*P* = 0.062), relative to the HFD group.

### 3.3. PY Extract Decreased the Level of SRA mRNA in Obese Adipose Tissue

We examined SRA, CD36, and LOX-1 gene expression in adipose tissue. HFD feeding significantly increased the mRNA levels of SRA, CD36, and LOX-1 (*P* = 0.001, *P* = 0.038, and *P* = 0.009, resp.) relative to the RCD group, and PY extract significantly suppressed the expression of the SRA gene, by 54% (*P* = 0.046 versus HFD group), but not that of the CD36 and LOX-1 genes (*P* = 0.245 and *P* = 0.235 versus HFD group) (Figure 2(b)).

### 3.4. Effect of PY Extract and Its Fractions on Scavenger Receptor Gene Expression in PMA-Stimulated THP-1 Cells

**Figure 3:**
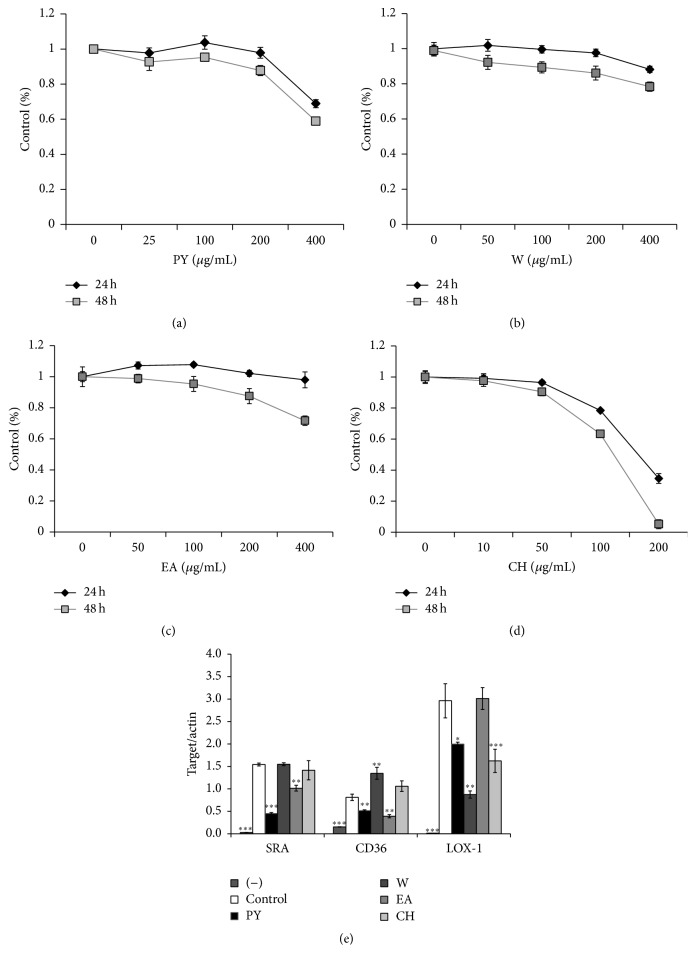
Effect of PY extract and its fractions on PMA-stimulated cell viability and scavenger receptor gene expression. ((a)–(d)) THP-1 cells were cultured with PMA (100 nM) for 24 h and 48 h. PY extract (a) and its aqueous (W), ethyl acetate (EA), and chloroform (CH) fractions were added into the media simultaneously with PMA. Cell viability was determined by reading the amount of colored formazan. (e) THP-1 cells were stimulated with PMA for 48 h, and PY extract (200 *μ*g/mL) or fractions (W: 100 *μ*g/mL, EA: 100 *μ*g/mL, CH: 10 *μ*g/mL) thereof were added into the media simultaneously with PMA. Gene expression levels were determined using qPCR. Target gene expression was normalized to that of actin. Data are expressed as means ± SD of three independent experiments. (−) PMA negative. ^*∗*^ *P* < 0.05, ^*∗∗*^ *P* < 0.01, and ^*∗∗∗*^ *P* < 0.005 versus cells treated with PMA only (control).

THP-1 cells are a suspension cell type that resembles circulating monocytes. PMA, a protein kinase C (PKC) agonist, stimulates THP-1 cells to differentiate into adherent macrophages and express scavenger receptors [13]. At the cellular level, PY extract and its water, ethyl acetate, and chloroform fractions decreased the number of attached THP-1 cells in a concentration- and time-dependent manner (Figures 3(a)–3(d)). We employed real-time quantitative PCR analysis to test the effects of 200 *μ*g/mL of PY extract and 100 *μ*g/mL of the water and ethyl acetate fractions. For the chloroform fraction, we chose 10 *μ*g/mL, the highest concentration at which cell viability greater than 90 percent was retained. Even in* in vitro* culture, SRA expression was suppressed in cells treated with PY extract to a greater degree than were CD36 and LOX-1 (Figure 3(e)). SRA gene expression was reduced to 30% of the level observed in control cells, while those of CD36 and LOX-1 were 64% and 68% of the levels observed in control cells, respectively. In fractionation guided by the inhibitory effect on the SRA and CD36 genes, the ethyl acetate fraction conferred inhibition of 35% and 50%, respectively, but showed no activity toward LOX-1. The aqueous fraction reduced the level of expression of LOX-1 by 70% but contrastingly enhanced that of CD36 by 65%. The chloroform fraction acted on LOX-1 alone.

### 3.5. Suppression of PMA-Stimulated ERK1/2 Activity by PY Extract and Its Ethyl Acetate Fraction

**Figure 4:**
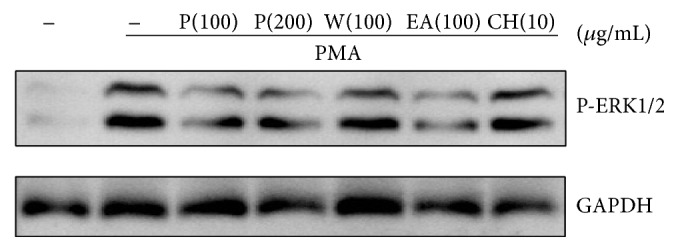
Effect of PY extract and its fractions on PMA-stimulated ERK1/2 activation. THP-1 cells were pretreated with PY extract or each fraction for 1 h and then stimulated with PMA for 30 min. GAPDH was used as an endogenous control. One representative western blot out of three experiments is shown. P: PY extract, W: water fraction, EA: ethyl acetate fraction, and CH: chloroform fraction.

SRA expression by PMA in THP-1 cells involves the Raf-MEK-ERK1/2 pathway [14, 15]. We analyzed whether PY extract and its fractions affect PMA-stimulated ERK1/2 phosphorylation. Suppression of ERK1/2 activity was observed in cells treated with PY extract or the ethyl acetate fraction (Figure 4).

### 3.6. HPLC and the Contents of Total Phenolics and Flavonoids

**Figure 5:**
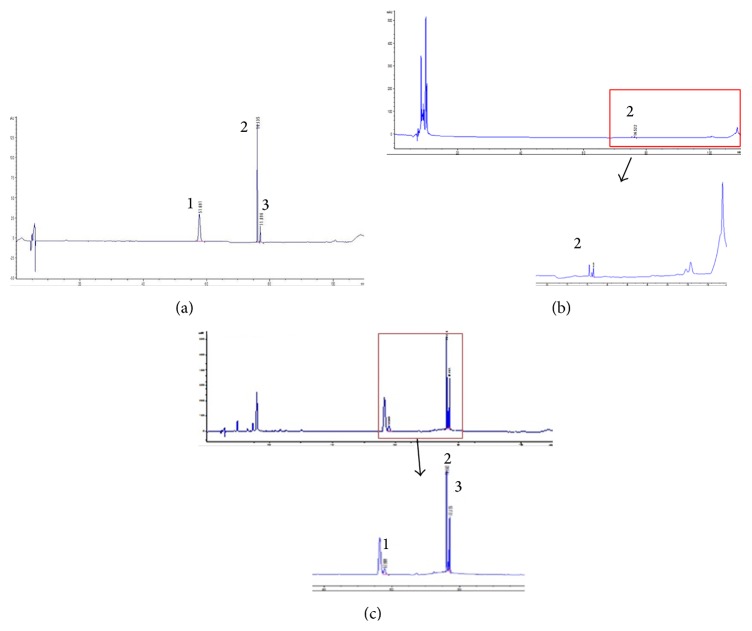
HPLC chromatogram of PY extract and of the chloroform fraction. (a) Standard mixture: peak 1: genistein; peak 2: sakuranetin; peak 3: prunetin. (b) PY extract (2 mg/mL). (c) Chloroform fraction (2 mg/mL).

We chose sakuranetin, prunetin, and genistein for quality control of PY extract. According to HPLC analysis, prunetin and genistein were detected in the chloroform fraction but not in PY extract (Figure 5). None of these compounds were detected in water or ethyl acetate fractions (data not shown). The regression equation of the three compounds and their correlation coefficients (*r* ^2^) were estimated based on the plots of the peak-area (*y*) versus concentration (*x*): prunetin, *y* = 4.3303*x* − 0.6793 (*r* ^2^ = 0.9999); genistein, *y* = 65.096*x* − 8.1315 (*r* ^2^ = 0.9999); and sakuranetin, *y* = 2.4284*x* − 0.3595 (*r* ^2^ = 0.9999). The amount of the three flavonoids identified in the chloroform fraction was in the order of prunetin (16.81 ± 0.5 mg/100 mg of fraction dry weight) > sakuranetin (9.08 ± 0.4 mg/100 mg) and genistein (3.29 ± 0.4 mg/100 mg). We determined the contents of total phenolics and flavonoids in each fraction using colorimetric methods. The highest concentration of total phenolic and flavonoid contents was observed in the ethyl acetate fraction (Table 1).

## 4. Discussion

Adipose cells store extra glucose and triglycerides and release them in the form of fatty acids and glycerol in times of need. They also act as endocrine cells that secrete adipokines, biomolecules that can affect other cells. Such adipokines include inflammatory chemokines and cytokines. TNF-*α*, which is mainly produced by macrophages and, to a lesser degree, by adipocytes in obese adipose tissue, can disrupt insulin/insulin-like growth factor signaling and contribute to insulin resistance [16]. Based on this, some anti-inflammatory therapies have been successful in improving insulin sensitivity. High doses of salicylate decrease fasting blood glucose in subjects with type 2 diabetes [17]. In addition, the use of a TNF-*α* neutralizing antibody ameliorated insulin resistance in patients with rheumatoid arthritis [18, 19]. Given this fact, obesity without inflammatory responses may be protective against the development of insulin resistance and type 2 diabetes [20]. PY extract significantly decreased these inflammatory markers in mice fed the HFD, although their body weight was not significantly altered. The reduction of elevated fasting blood glucose levels by PY extract might be accounted for by its inhibitory effect on these inflammatory cytokines.

Hypertrophy of adipocytes in obese adipose tissue leads to their eventual death, and most macrophages in the tissue take residence around moribund adipocytes, scavenging dead cells and other cellular debris [21]. Therefore, it is not surprising to observe that the upregulation of scavenger receptor genes occurred in adipose tissue macrophages. There are conflicting reports as to the role of scavenger receptors in experimental obese mice. While SRA-deficient mice fed a HFD exhibited increased levels of fasting blood glucose and aggravated inflammatory responses, CD36 knockout mice fed a HFD showed reduced adipose tissue mass, increased insulin sensitivity, and decreased inflammatory markers [22, 23]. The authors above showed that SRA in obese adipose tissue promoted the polarization of M2 macrophages, an alternatively activated population whose phenotype is anti-inflammatory and efficient in the clearance of cellular debris, as opposed to the classically activated type, M1 macrophages, which are producers of proinflammatory cytokines [22, 24]. On the other hand, obese nondiabetic humans exhibited increased expression of SRA, LOX-1, and CD36 in subcutaneous adipose tissue [10]. In particular, SRA gene expression was strongly correlated with the level of insulin [10]. Although SRA and other scavenger receptors are regarded as necessary for the differentiation of M2 macrophages, the pro- and anti-inflammatory actions of scavenger receptors are context-dependent [7]. It is possible that mice deficient in SRA may be impaired in removal of lipid wastes, which can trigger sterile inflammation. Expression of SRA is induced in response to high glucose without overt inflammatory responses. SRA upregulation was observed both in peritoneal macrophages from streptozotocin-treated diabetic mice, which exhibited an extremely high level of lipids and glucose and in peripheral blood mononuclear cells from hyperlipidemic patients [9, 25]. The downregulation of SRA gene expression in obese adipose tissue by PY extract may reflect a reduction in the levels of blood glucose or lipids.

Reactive oxygen species (ROS) generation and PKC activation is implicated in the enhanced expression of scavenger receptors in macrophages exposed to high glucose [9, 26]. ERK1/2 activation is downstream of PKC-stimulated ROS production [14]. Treatment of PMA-stimulated THP-1 cells with PY extract inhibited scavenger receptor gene upregulation and ERK1/2 activation. It is possible that antioxidant components within the PY extract contribute to its inhibitory effects on ERK1/2 activity and monocyte differentiation via a modulation of ROS generation.

We further investigated to determine the active fraction of PY extract. The chloroform fraction was highly toxic to cells relative to the other fractions and required using a dose an order of magnitude lower than that of the water or ethyl acetate fraction. We did not clarify which component(s) within the extract were responsible for the observed toxicity of the chloroform fraction. None of the fractions showed an activity similar to that of the unfractionated PY extract. Each fraction acted on different genes. The inhibitory effect of the ethyl acetate fraction on SRA expression and ERK1/2 activity may be associated with its high concentration of total phenolic and flavonoid contents.

Absorbed phytochemicals including flavonoids undergo extensive first-pass metabolism in the intestine and liver and are found in the plasma as glucuronides, sulfates, and methylated metabolites [27]. Furthermore, some flavonoids exist in their glycosides or other polymers within a crude extract and require hydrolysis by the intestinal enzymes and colonic microflora. It is generally believed that unconjugated flavonoids exist in the range of less than 5% in plasma, accounting for their limited bioavailability [28]. Such* in vivo* concentrations are much lower than those needed to achieve* in vitro* biological activities [27]. However, it cannot be ruled out that metabolites of flavonoids are actually the bioactive molecules* in vivo*. The PMA-stimulated THP-1 cell model employed by our investigation reflects merely a part of the many biological events in diet-induced obesity. Therefore, it may be an overstatement to conclude that the bioactive components of PY extract* in vivo* can be found in the fraction showing activity in the modulation of scavenger receptor expression in THP-1 cells.

We quantified prunetin, genistein, and sakuranetin in the chloroform fraction. Percent recovery of prunetin, genistein, and sakuranetin was determined to be 0.42%, 0.22%, and 0.08% of dry weight of PY extract. This estimation was based on the yield of chloroform fraction. Considering that glycosides of prunetin and genistein were also found in PY extract, more than these amounts would be bioavailable [29, 30]. With regard to bioavailability, data on prunetin is not available. In the case of genistein, absorption is good but the plasma level of unconjugated genistein is less than 10% while that of total genistein (unconjugated and conjugated genistein) is close to 90% in rodents, indicating low oral bioavailability [31]. In addition, prunetin can be converted to genistein by the hepatic P450 enzymes via a demethylation pathway [32]. In several studies, consumption of genistein was demonstrated to confer an antiobesity effect and downregulate hepatic SRA expression in mice fed a HFD [33–35]. Intranasal treatment of sakuranetin attenuated* in vivo* airway allergic inflammation [36]. As yet, we do not know whether sakuranetin is a phytochemical with potential antiobesity properties. Overall, the presence of prunetin and genistein and other unidentified phytochemicals may help to explain some of the inhibitory effects of PY extract on genes associated with obesity-induced inflammation and hyperglycemia.

## 5. Conclusions

The current study demonstrated that PY extract, despite its failure to prevent an increase in body weight, was able to suppress obesity-associated inflammatory responses and fasting blood glucose. We suggest that the downregulation of SRA by PY extract mediates a reduction in levels of blood glucose and lipids, in diet-induced obesity.

## Figures and Tables

**Table 1:** The contents of total phenolics and flavonoids.

	Water	Ethyl acetate	Chloroform
Total phenolics (mg GAE/g)	8,803 ± 80	64,674 ± 887	248 ± 9

Total flavonoids (mg CTE/g)	8,770 ± 794	13,840 ± 1210	9 ± 0.2

The contents of total phenolics and flavonoids were measured as described in Section 2. Results are expressed as means ± SD, *N* = 3.
